# Complications of Infective Endocarditis: A Case Report of Intracerebral Hemorrhage Exacerbated by Enoxaparin

**DOI:** 10.7759/cureus.61235

**Published:** 2024-05-28

**Authors:** Steven Pham, Benjamin Heigle, Connor Gibbs, Wubet G Gebrehiwot, Prabhneet Pannu

**Affiliations:** 1 Internal Medicine, White County Medical Center, Searcy, USA; 2 Emergency Medicine, White County Medical Center, Searcy, USA

**Keywords:** vancomycin, stroke, enoxaparin, intracerebral hemorrhage, endocarditis

## Abstract

Infective endocarditis (IE) can cause life-threatening intracerebral hemorrhage via the transformation of an embolic ischemic stroke. Navigating anticoagulant therapy for IE patients is challenging due to this risk. Hospitalized patients often receive anticoagulation to minimize venous thromboembolism (VTE). Those at higher VTE risk may require full anticoagulation, particularly if there is an initial suspicion of a blood clot. A timely IE diagnosis is crucial but is often delayed during inpatient stays, with the patient potentially already on anticoagulants for other conditions. Our case discusses a hemorrhagic stroke in a patient with IE while receiving therapeutic enoxaparin. Clinical signs and symptoms, echocardiographic findings, laboratory workup and microbiological data, and possibly other imaging techniques such as cerebral magnetic resonance imaging (MRI) need to be employed in a timely manner in determining endocarditis as a cause of stroke.

## Introduction

Endocarditis is one of the major causes of cardiac embolism and carries high morbidity and mortality rates [1]. A study published in 2020 found that the yearly occurrence of endocarditis ranged from three to 10 cases per 100,000 individuals, with mortality rates remaining high at 30% within 30 days [2]. Three main forms of endocarditis have been described: infective endocarditis (IE), nonbacterial thrombotic endocarditis (NBTE), and Libman-Sacks endocarditis (LSE). Infective endocarditis is the most common form of endocarditis, while malignancy is often associated with NBTE, and patients with systemic lupus erythematosus and antiphospholipid syndrome frequently exhibit LSE [3]. Regrettably, the diagnosis of embolism is often delayed, underscoring the urgency for timely recognition and intervention [1]. 

Approximately 20%-50% of patients experience embolic events as a result of the migration of infectious material into the arterial circulation [4]. Notably, the brain is a frequent target for embolism, potentially complicating treatment strategies. The majority of cerebral emboli in IE patients are silent, which further highlights that MRI is needed to identify stroke [1]. Among neurologic complications in IE, stroke and transient ischemic attacks stemming from the occlusion of cerebral arteries contribute to 40%-50% of cases, surpassing other complications such as meningitis or meningeal reaction, brain abscess, mycotic aneurysm, and intracranial hemorrhage [1]. The mainstay of treatment for IE is systemic antibiotic therapy. However, in some patients, cardiac surgery is warranted [5]. A timely surgical intervention is crucial in the management of infective endocarditis, serving as a cornerstone to mitigate the risk of embolization [5]. When neurologic embolization occurs, it can exacerbate the prognosis and introduce uncertainties regarding potential deterioration or the likelihood of hemorrhagic conversion [5]. Although there have been improvements in diagnostics and therapy, both diagnosis and treatment remain difficult, and mortality in IE patients remains high. 

Neurological complications often correlate with an unfavorable prognosis [6, 7]. The neurological symptoms include but are not limited to, ischemic stroke, intracranial hemorrhage, infectious intracranial aneurysm, meningitis, and brain abscesses [8]. The highest risk of stroke occurs at the time of diagnosis and the initiation of antibiotic treatment, diminishing significantly after two weeks from the initiation of antibiotics [9]. Among neurological complications, intracerebral hemorrhage is the most fatal, with an incidence ranging from approximately 7% to 27% in IE cases [10]. In patients with ischemic stroke secondary to IE, intravenous (IV) thrombolysis is contraindicated. However, the diagnosis of IE is frequently not known upon admission and is instead established later during the inpatient stay, introducing complexities in management.

## Case presentation

A 76-year-old female with a history of type 2 diabetes mellitus, essential hypertension, chronic kidney disease stage 3, paroxysmal atrial fibrillation, coronary artery disease with bypass surgery, and aortic stenosis with aortic valve replacement presented with the chief complaint of pleuritic chest pain that started two days prior to admission. She had bypass surgery around 20 years ago, along with the valve replacement; she did not remember the exact time. She had been taking amiodarone 200 mg daily and metoprolol succinate 25 mg daily for her heart condition. The initial workup revealed these abnormalities: white blood cell count: 20.8 th/uL with 87.2% neutrophils, blood urea nitrogen (BUN): 46.0 mg/dL, creatinine: 1.9 mg/dL, procalcitonin: 3.78 ng/mL, and D-dimer: 3.22 mg/L (Tables 1-2). Due to concerns about pneumonia, she was started on intravenous (IV) ceftriaxone (2 grams). Given the elevated D-dimer, there was suspicion of pulmonary embolism, so the patient was started on therapeutic enoxaparin. A transthoracic echocardiogram showed a left ventricular ejection fraction of 65%-70% with normal prosthetic aortic valve function. Her blood cultures grew *Staphylococcus aureus* on both bottles, and vancomycin was added. Because the patient had chronic kidney disease and her kidney was also worsening from baseline on admission, she was not a candidate for a CT pulmonary angiogram. Therefore, a ventilation-perfusion (V/Q) scan was performed, which ruled out pulmonary embolism. Due to her renal function, the frequency of the therapeutic enoxaparin dose was changed from twice a day to daily. Because of this change, the patient received two doses of therapeutic enoxaparin before pulmonary embolism was ruled out. Enoxaparin was later switched to prophylactic heparin, 5,000 units subcutaneously every 12 hours. 

**Table 1:** Basic metabolic panel (BMP) during the patient's stay at our facility

	Sodium (mmol/L); range: 137-145	Potassium (mmol/L); range: 3.5-5.0	Chloride (mmol/L); range: 98-107	Carbon dioxide (mmol/L); range: 22-30	Blood urea nitrogen (mg/dL); range: 7.0-17.0	Creatinine (mg/dL); range: 0.7-1.2	Glucose (mg/dL); range: 65-105
Day 1	137	4.9	104	23	46	1.9	146
Day 2	137	4.6	104	23	52	1.8	129
Day 3	137	4.2	106	24	39	1.2	104

**Table 2:** Complete blood count (CBC) during the patient's stay at our facility

	White blood cells (th/uL); range: 4.5-11.0	Red blood cells (mil/uL); range: 4.2-5.4	Hemoglobin (g/dL); range: 12.0-16.0	Hematocrit (% ); range: 37.0-47.0	Neutrophils (%) ; range: 40.0-80.0
Day 1	20.8	3.51	11.6	34.9	87.2
Day 2	14.6	3.47	11.4	35.0	87.8
Day 3	12.6	3.47	11.4	34.5	87.8

**Figure 1:**
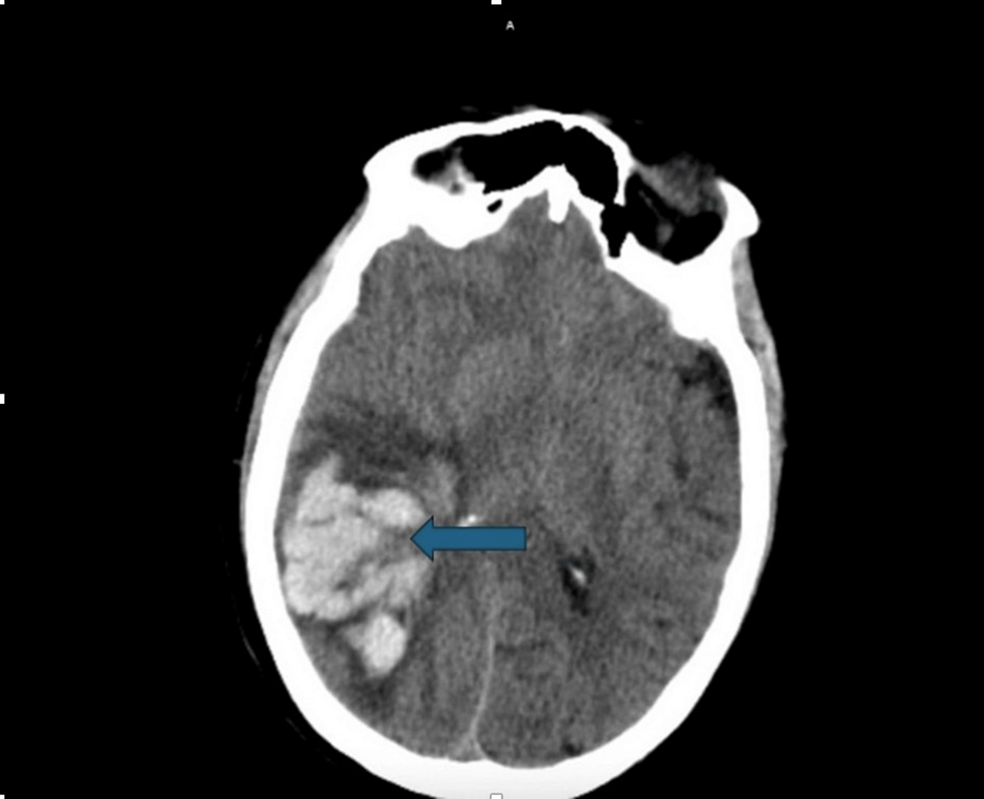
A CT scan of the head shows a large intraparenchymal hemorrhage measuring 6.8 x 4.8 x 7.4 cm.

The next morning, when we evaluated the patient, she was drowsy but alert and oriented to herself, the people around her, and the place and time. She complained of a diffuse headache, but the headache had been persistent for the past several days. However, two hours later, we were contacted by the nursing staff with the concern that the patient had new left-sided neurologic deficits. Her last known well time was around an hour prior to us being contacted. The physical exam at the bedside was worrisome for left-sided facial asymmetry, slurred speech, and weakness in the left upper and lower extremities. An immediate computed tomography (CT) of the head without contrast showed a large intraparenchymal hemorrhage measuring 6.8 x 4.8 x 7.4 centimeters (cm) (Figure 1). There was also surrounding vasogenic edema with a mass effect on adjacent parenchyma and an approximately 6 mm right-to-left midline shift. She was started on a diltiazem drip to control blood pressure prior to transfer to another facility for evacuation of the hematoma due to the lack of an in-house neurosurgeon at our facility. Before the evacuation, she was also given phenytoin and tranexamic acid (TXA) in an attempt to potentially limit hematoma expansion.

The patient signed consent for us to obtain medical records from the outside facility where she was transferred. At an outside facility, a repeat CT head was obtained after the evacuation of the hematoma, which showed a stable appearance of the right occipital temporal convexity cavity with 4 mm of right-to-left subfalcine herniation. Another echocardiogram was performed, which showed vegetation on the mitral valve and moderate to severe mitral regurgitation. She developed sepsis secondary to bacteremia from the mitral valve endocarditis and multifocal pneumonia. Her antibiotics were escalated to vancomycin and cefepime. Following the hematoma evacuation, she, unfortunately, remained intubated for the documented reason that she experienced worsening respiratory failure. The documentation from the outside facility showed that the physicians who took care of the patient had a thorough discussion with the family to move toward comfort-directed care. The patient was compassionately extubated and expired after.

## Discussion

Infective endocarditis poses a substantial risk of vascular complications, including cerebral embolism, intracerebral hemorrhage, and aneurysm, all of which correlate with high mortality [11]. While anticoagulation serves as a fundamental management strategy for thromboembolic complications, its application remains controversial and challenging for IE patients [12]. Previous studies have indicated that anticoagulant treatment does not effectively mitigate the risk of ischemic stroke in IE patients, underscoring that IE alone may not be a sufficient indication for anticoagulation [12]. However, the current guidelines on managing stroke complications from IE are still lacking, offering limited specific recommendations on anticoagulation.

The occurrence of stroke in individuals with IE is estimated to be around 10%, stemming from either embolic infarction or hemorrhage [13]. Ischemic stroke tends to be more prevalent than hemorrhagic events [13]. Intracranial hemorrhages during uncontrolled infection are often attributed to septic emboli inducing pyogenic arteritis and mycotic aneurysm [14, 15]. This is often attributed to the delayed initiation of antibiotic therapy [16, 17]. Several case studies highlight that the majority of neurological complications occur before antibiotics are initiated. Therefore, the crucial intervention to prevent further strokes is the timely administration of antibiotic therapy [16, 17]. 

The composition of the vegetation in ischemic stroke includes platelets, fibrin, microorganisms, inflammatory cells, and leaflet disruption [18]. Given this composition, fibrinolytic therapy has the potential to assist in the resolution of emboli containing fibrin from vegetation or new fibrin that may form post-embolization [14]. However, the safety and efficacy of thrombolysis in infective embolic strokes are still not fully understood. For patients with IE, thrombolytic therapy for acute ischemic stroke is not recommended [19]. In a retrospective study by Walker et al. that involved 18 cases of IE-related stroke, those who received thrombolysis experienced a high mortality rate of 75% [17]. However, another study reported four cases where patients receiving thrombolysis for ischemic stroke secondary to IE exhibited favorable outcomes [7]. Additionally, in another report, mechanical thrombectomy with or without adjuvant thrombolytics demonstrated positive outcomes in seven out of 15 cases of IE-related stroke [20]. These studies show conflicting results, and whether or not thrombolytics is indicated in patients with IE-associated stroke remains debatable [12]. 

While anticoagulation is crucial for managing venous thromboembolism, its application in IE remains controversial due to the high risk of vascular complications. Though anticoagulation theoretically reduces embolic stroke risk by targeting blood products in cardiac vegetation, there is insufficient evidence to guide decisions in IE and stroke. In our case, therapeutic enoxaparin was administered due to concerns about PE, not IE. Whether anticoagulation worsens hemorrhagic stroke in our patient remains uncertain, as evidence is inconclusive. Further studies are needed to address this debate.

## Conclusions

Despite carrying a high mortality rate, IE is frequently overlooked. One of the serious complications of IE is intracerebral hemorrhage, which consequently may arise earlier than the diagnosis is established. Our patient was initially administered anticoagulation due to concerns about pulmonary embolism before the initiation of antibiotics and even before the diagnosis of stroke was established. There is no established consensus or guideline regarding the initiation of anticoagulation in such patients, making the decision inherently challenging. Cardiac vegetations contain blood products like platelets and fibrin, suggesting that anticoagulation might reduce the likelihood of embolic strokes. However, IE can lead to mycotic aneurysms and widespread cerebral microhemorrhages, potentially increasing the risk of intracerebral hemorrhage with anticoagulation. Additional studies are warranted to investigate the impact of anticoagulation on these patients.
